# Towards the measurement of food literacy with respect to healthy eating: the development and validation of the self perceived food literacy scale among an adult sample in the Netherlands

**DOI:** 10.1186/s12966-018-0687-z

**Published:** 2018-06-18

**Authors:** Maartje P. Poelman, S. Coosje Dijkstra, Hanne Sponselee, Carlijn B. M. Kamphuis, Marieke C. E. Battjes-Fries, Marleen Gillebaart, Jacob C. Seidell

**Affiliations:** 1 Department of Human Geography and Spatial Planning, Faculty of Geosciences, Utrecht University, Princetonlaan 8a, 3584 CB Utrecht, The Netherlands; 2 Amsterdam Public Health research institute, Department of Health Sciences, Faculty of Earth and Life Sciences, VU-University Amsterdam, De Boelelaan 1085, 1081 HV Amsterdam, the Netherlands; 3 Department of Social Health and Organizational Psychology, Utrecht University, Utrecht, The Netherlands; 4 Department of Interdisciplinary Sciences, Utrecht University, PO Box 80140, 3508 TC Utrecht, The Netherlands; 5 Department of Health and Nutrition, Louis Bolk Institute, Kosterijland 3-5, 3981 AJ Bunnik, The Netherlands

**Keywords:** Food literacy, Questionnaire, Eating behavior, Food consumption, Health, Impulsiveness, Self-control

## Abstract

**Background:**

Food literacy refers to the capability to make healthy food choices in different contexts, settings and situations. The aim of this study is to develop and validate the self-perceived food literacy (SPFL) scale, to assess individuals’ level of food literacy, including a knowledge, skills and behavior to plan, manage, select, prepare and eat food healthfully.

**Methods:**

An initial set of 50 items for the SPFL scale were generated based on expert insights and literature. A cross-sectional online survey was conducted among a sample of Dutch adults (*n* = 755) in order to determine convergent, divergent and criterion validation against psychosocial variables that were expected to correlate with food literacy (self-control, impulsiveness) and against the expected outcome of high food literacy, namely healthy food consumption. Principal Component Analyses (PCA), Pearson correlation tests and linear regression analyses were conducted. The capacity to distinguish of the SPFL scale was determined by comparing SPFL scores of the general population with that of a sample of dieticians (*n* = 207).

**Results:**

The participants in the general sample had an average age of 44.8 (SD:16.1), the majority were women (90.7%), they had a healthy weight (61.4%) and were highly educated (59.1%). Of the initial 50 items, 29 items remained after PCA and reflected eight domains of food literacy. SPFL was positively correlated with self-control (*r* = 0.51, *p* = <.001) and negatively with impulsiveness (*r* = − 0.31, *p* = <.01). Participants with higher levels of food literacy reported a significantly higher frequency of fruit consumption (≥5 times/week), vegetable consumption (≥5times/week) and fish consumption (≥1times/week) and consumed larger portions of fruit (≥2pieces/day) and vegetables ≥200 g/day) in comparison with participants who had lower levels of food literacy. Dieticians had slightly higher scores on SPFL than general adults (B = 0.08, SE = 0.03, *t* = 2.83, 95%-CI = 0.03 to 0.14).

**Conclusions:**

The 29 item SPFL scale is a validated, expert-based and theory-driven tool for measuring self-perceived food literacy with respect to healthy eating among adults. Higher levels of food literacy were associated with more self-control, less impulsiveness and healthier food consumption. Additional research is needed to validate the SPFL scale in different populations (different age groups, socioeconomic groups, male populations) and in different contexts.

**Electronic supplementary material:**

The online version of this article (10.1186/s12966-018-0687-z) contains supplementary material, which is available to authorized users.

## Background

Unhealthy food habits, defined as a diet high in refined carbohydrates, sodium, saturated fat and calories, are associated with an increased risk of obesity and other non-communicable diseases (NCDs) and premature mortality [1, 2]. Food consumption is a complex behavior which is affected by multiple factors, ranging from individual and social to environmental determinants [3]. The current food environment promotes ready-to-eat, low-cost, highly processed foods which are energy dense and nutrient poor [4]. This makes navigating the food environment in a healthy way a challenge for many people. In addition, particular circumstances that are common in today’s Western everyday life - e.g. being rushed, having too little sleep and experiencing psycho-social stress - make people even more vulnerable to making unhealthy food decisions [5–7]. Only a small proportion of the population manage to make healthy food choices and achieve recommended nutrition guidelines. For example, only 15% of the Dutch adult population fulfil the fruit and vegetable recommendations of the Health Council of the Netherlands [8].

Those individuals who possess the capability to make everyday healthy food choices in different contexts, settings and situations are considered to be ‘food literate’. Food literacy has emerged as a distinct form of the more general concept of ‘health literacy’ [9]. Health literacy can be defined as ‘*people’s knowledge, motivation and competences to access, understand, appraise and apply health information in order to make judgments and take decisions in everyday life concerning healthcare, disease prevention and health promotion to maintain and improve quality of life throughout the life course*’ [10]. Although several definitions of food literacy exist [11–13], one of the most comprehensive ones is ‘*the scaffolding that empowers individuals, households, communities or nations to protect diet quality through change and strengthen dietary resilience over time. It is composed of a collection of interrelated knowledge, skills and behaviors required to plan, manage, select, prepare and eat food to meet needs and determine food intake’* [14]*.* In the Netherlands, food literacy focuses on the individual domain and emphasises ‘people’s knowledge of healthy eating and their capability to purchase and prepare healthy foods’ [15]. These definitions clearly emphasize that food literacy is more than just knowledge and that it is a comprehensive concept of a variety of determinants needed to eat healthily. Higher food literacy is conceptually related to better nutritional quality, health and well-being [12, 16–20].

Attention for food literacy and public health programmes targeting food literacy have been on the increase during the past decade in both society and academia [21–24]. In 2016 the Dutch Ministry of Economic Affairs and the Ministry of Health, Welfare and Sport introduced the ‘Jong Leren Eten’ programme (translation: ‘Learning how to eat early on’) and allocated approximately 6 million euros to extend and improve the adoption of nutrition education programmes in schools (jonglereneten.nl). In academia research on this topic has also increased, as indicated by only 22 Google Scholar hits on a simple search task for the term ‘food literacy’ in 2006, which rose to 95 hits in 2011, and 321 hits in 2016. With the increased societal and academic efforts aimed at improving food literacy, it is important to know and monitor over time whether people have become more food literate as a result of these initiatives. It is also important to understand whether food literacy, as conceptually proposed, is indeed empirically associated with healthy food consumption.

Although various questionnaires have been used to determine certain elements of food literacy (e.g. preparation skills, understanding of nutrition fact labels) [14], eating competence (including contextual food skills) [25] and food literacy elements as derived from a health literacy scale [26, 27], a measure to assess individuals’ level of food literacy, including the collection of interrelated knowledge, skills and behaviors to plan, manage, select, prepare and eat food healthfully, does not yet exist.

This is an important research gap, both in observational as well as intervention research. For example, it is of interest to identify individual, social or contextual circumstances that interfere with food literacy. Regarding the latter, we see that nutrition education programmes are mainly evaluated based on their effects on specific behavioral determinants (e.g. attitudes or self-efficacy towards healthy eating) but not on people’s overall capability to plan, manage, select, prepare and eat foods healthfully.

Therefore, the aim of this study is to develop and validate a self-perceived food literacy scale with respect to healthy eating among a sample of the Dutch adult population, and to explore associations with food intakes.

## Methods

### Study design

A mixed-method approach was used to develop the self-perceived food literacy scale (SPFL scale) comprising three steps. First, items of the SPFL scale were developed, based on expert insights and existing literature (Aug-Nov 2016). Second, a cross-sectional online survey was conducted in December 2016 and January 2017 among a sample of Dutch adults, in order to: 1) determine the component structure and reliability of the SPFL scale; 2) validate the scale against psychological constructs which are well known for their positive (self-control) and negative (impulsiveness) correlation with healthy food consumption (convergent and divergent validity), and 3) explore associations of the SPFL scale with (un)healthiness of food consumption (criterion validity). Third, the SPFL scale’s capacity to distinguish between subgroups with a higher or lower food literacy was determined and the same online survey was used on a sample of registered dieticians in January 2017. We hypothesized that registered dieticians would, on average, have higher levels of food literacy compared to those recruited from the Dutch adult population. The study followed the standards of the ethical committee of the Faculty of Social Science of Utrecht, the Netherlands.

### Scale development

In the first stage of the SPFL scale development an expert meeting was organised with health professionals and academics working in the field of food literacy (*n* = 10). During this meeting the experts were asked to provide input for specific scale items concerning knowledge, skills and behaviors related to food literacy. To guide theory-driven item development we presented a grid which combined the domains of food literacy as expressed by Vidgen et al. (planning/managing, selecting, preparing, eating) [28] on the Y axis, and the four aspects of health literacy as defined by Sørensen et al. (namely access, understand, appraise and apply) on the X axis. [10, 29]. In couples experts discussed and wrote down necessary scale topics for each aspect of the food literacy domains with respect to healthy eating. The experts also defined food literacy practices that were not necessarily covered by the food literacy domains. After that a plenary discussion was held of the experts’ input and then refined until consensus was reached.

In a second stage existing questionnaires covering different food literacy domains were gathered via scholar google and educational nutrition programme evaluations. Although numerous questionnaires exist, items of some surveys were specifically taken into account as these concerned similarities with certain food literacy domains [30–36] Based on scale input of the expert meeting and the existing questionnaires, a very comprehensive list of 144 items relating to food literacy was compiled. This longlist of food literacy items was sent to the experts involved in the meeting, as well as to an additional group of Dutch experts (academics, dieticians and health professionals) in the field of food consumption and health literacy (*n* = 17). To prevent the risk of steering the questionnaire in a particular direction according to our own preferences, we decided not to reduce the list with 144 items before sending it for feedback. The experts were asked via email to provide written feedback on the longlist by highlighting important items, adding missing items or topics, deleting or merging double items or non-food literacy items and emphasizing unclear items. Based on the input, most items were deleted. The majority were deleted because they reflected the same food literacy component. Some items were also deleted because they reflected another domain which is important for healthy eating (e.g. motivations, self-estimation) or indicated a practicality that was not necessarily expected to translate into healthy food consumption (e.g. I know the average price of foods, I find it important to set the table nicely when having dinner). Based on the input of all experts, items were deleted and revised. Subsequently, three researchers (MP, CD, JS) critically revised the list and created a final scale in which a total of 50 items remained. This questionnaire was translated by a Dutch linguistic company to ‘B1’ level which is generally understood by 95% of Dutch people. Moreover, items that consisted of a statement for which respondents would have to indicate their level of agreement (e.g. “*I am able to prepare vegetables in a variety of ways”)* were rephrased into questions (“*Are you able to prepare vegetables in a variety of ways?”*), as the latter is assumed to be easier. The scale was pilot tested among a small convenience sample (*n* = 10) and a few minor amendments were made to improve its comprehensibility and readability.

### Recruitment and participants

**Fig. 1:**
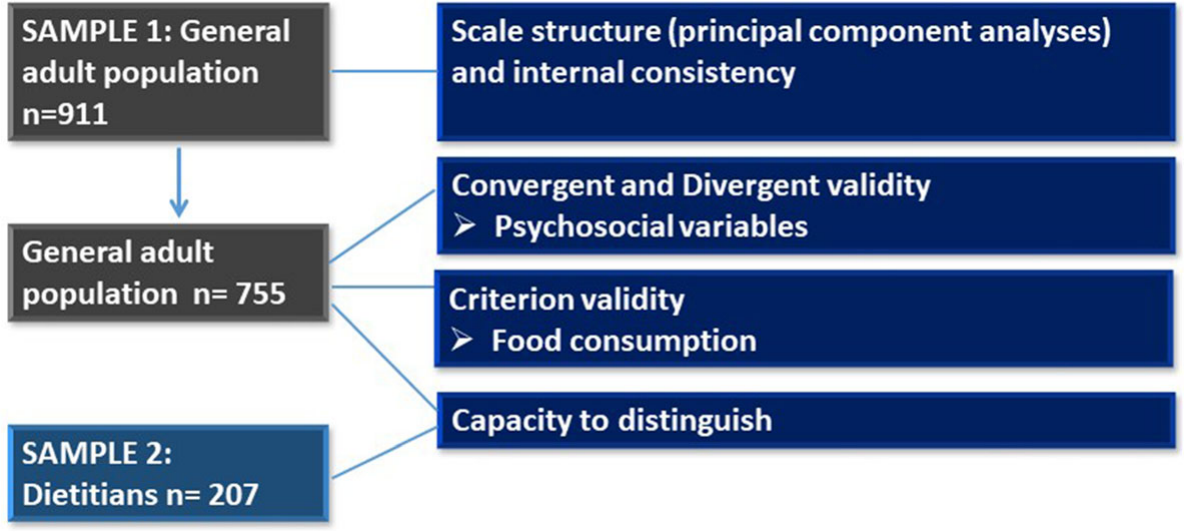
Participant flow in sub analyses to develop and validate the Self-Perceived Food Literacy scale. Footnote: of the initial 911 participants of the general population (that participated in the scale structure and reliability analyses), 755 participants completed the whole questionnaire and were included in the analyses required for validation. In total 207 dieticians also completed the questionnaire

Two study samples were recruited to fill out the same online survey. First, Dutch adults were recruited via the Facebook page and Twitter account of The Netherlands Nutrition Centre, an independent organization funded by the Dutch Ministry of Economic Affairs and the Ministry of Public Health, Welfare and Sport (www.voedingscentrum.nl/nl/service/english). A short message was used to ask adults to complete an online questionnaire about dietary behavior. A total of 911 participants completed all 50 items of the SPFL scale and were included in the analyses to determine the scales’ structure and its reliability. Participants that completed the entire online survey (*n* = 755, 83%) were included in the analyses to determine convergent, divergent and criterion validity and the scales’ distinctive capacity (Fig. 1).

Second, registered dieticians, members of the Netherlands Association of Dieticians (NAD), were also invited to complete the online survey. They were recruited via a monthly NAD newsletter which invited them to fill out the online questionnaire for scientific research purposes with respect to food literacy. Before starting the questionnaire, the content of the questionnaire was explained and the participants agreed that their answers would be used in scientific research. A total of 207 dieticians completed the full questionnaire and were included into the analysis to determine the capacity to distinguish of the questionnaire.

### Measures

The online survey consisted of items relating to demographic characteristics, the 50 item SPFL scale, psychosocial constructs that have typically been associated with (un)heathy eating (self-control, impulsiveness) and a food frequency questionnaire for six product categories.

#### Demographic characteristics

Age, sex, highest attained educational level (lower (‘those with less than secondary school or an A level certificate’), middle (‘those with A levels or Dutch A level equivalent (VWO) graduation certificate’) and higher (‘those with polytechnic or university degrees’)), ethnicity, height and body weight (to determine body mass index (BMI)) were self-reported. Participants could select ‘private’ if they did not want to share this information.

#### Food literacy

The preliminary SPFL scale consisted of 50 questions covering a wide range of food literacy practices with respect to planning/managing, selecting, preparing and eating practicalities. For example, ‘*Can you cook vegetables in a variety of ways*?’ or ‘*Do you buy healthy food, even if it is more expensive*?’. Response options included a 5-point Likert scale (1= ‘not at all/never’ to 5 = ‘yes/always’). Negative items were reversed, indicating that the higher the score, the higher food literacy is.

#### Self-control and impulsiveness

Self-control and impulsiveness, psychosocial variables known for their positive or negative association with healthy food consumption, especially when prompted by unhealthy options within the environment [37–39], were included in this study for convergent and divergent validation purposes. Self-control is the capacity to alter or override dominant response tendencies [40, 41] and the ability to regulate, thoughts and emotions [42, 43]. Self-control is both positively associated with healthy food consumption [44] and negatively associated with unhealthy food consumption [45]. Impulsiveness is characterized by displaying behavior with little or no forethought, reflection or consideration of the consequences [46]. Higher levels of impulsiveness are associated with higher energy intakes [47] and linked to unhealthy food choices [48], larger sensitivity to the selection of unhealthy foods in the contexts of unhealthy food environments [49] and a tendency to overeat, in response to external stimuli like advertisements and promotions [50]. In this study self-control was measured by means of the Brief Self-Control Scale (BSCS), a validated 13 item scale (e.g. ‘*I have a hard time breaking bad habits*’), which were answered on a 5-point Likert scale (1= ‘not at all like me’ to 5= ‘very much like me’) (Tangney et al., 2004). Impulsiveness was measured by the validated Abbreviated Baratt Impulsiveness Scale (ABIS), composed of 13 items (e.g. ‘*I plan tasks carefully*’), which were answered on a 4-point Likert scale (1= ‘rarely / never’ to 4= ‘almost always / always’) [51].

#### Food consumption

Food consumption was determined by means of a food frequency questionnaire (FFQ) which focused on the frequency -by number of days per week- that participants consumed fruit, vegetables, fish, sugar sweetened beverages (SSB), ‘large’ unhealthy snacks (e.g. pizza slice, piece of pie) and ‘small’ unhealthy snacks (e.g. biscuit, candy). Nine response items were presented (1 = ‘never/seldom’ to 9 = ‘everyday’). Questions were also asked about the amount of these products consumed per day (in portions or glasses in case of SSB), except for fish, and these produced seven response items (1 = ‘< 1 portion/day’ to 7 = ‘> 5 portions/day’). The FFQ method is widely used and has been shown to be a valid, inexpensive and easy tool to provide a reasonably accurate ranking of intake to identify persons with a low vs. high intake [52–54].Statistical analyses.

All analyses were conducted using IBM SPSS statistics 23.0.

#### Principal component analyses

In order to determine the component structure of, and reduce the items on, the preliminary 50 item SPFL scale, an exploratory principal components analysis (PCA) with oblique rotation was used. After executing PCA, the Kaiser-Meyer Olkin (KMO) measure of sampling adequacy (required to be > 0.6) and the Bartlett’s Test of Sphericity (required to be statistically significant, *p* < 0.05) were determined. In addition, correlations between individual items of the scale were verified for not correlating above 0.9 (in order to avoid multicollinearity problems). Subsequently, the extracted number of components of the SPFL scale were explored based on the scree plot and eigenvalues based on the output. A parallel analysis using Monto Carlo PCA was also conducted to determine the random set of simulated eigenvalues (variables = 50; subjects = 911; replications = 100). Eigenvalues in the actual dataset were not allowed to exceed the eigenvalues produced in the simulated dataset. Next, PCA was conducted again in order to specify the appropriate number of components based on the scree plot and parallel analyses. Based on these outcomes commodities were checked and items with low loadings (< 0.3) were excluded from the analyses. Subsequently, PCA was conducted again. In this final phase item-loadings on each component were checked. First of all items that did not load on one of the components, or had low loadings on multiple scales were removed and PCA was run again. Subsequently, items with a component loading below 0.5 were removed from the analyses and PCA with the remaining items was run a final time. The explained variance of the overall scale was determined for each PCA.

#### Internal consistency

After conducting PCA the internal consistency for the overall scale and for each component within the scale was determined by means of the Cronbach’s α. A value of Cronbach’s α above 0.8 was considered to be an indicator of good reliability and Cronbach’s α above 0.7 was defined as an indicator for an adequate reliability. Factors with Cronbach’s α values below 0.4 indicated a low internal consistency.

#### Convergent and divergent validity

In order to explore convergent and divergent validity of the SPFL scale, Pearson correlations between the SPFL and impulsivity and self-control were calculated. Correlations had to be below 0.9 (large correlation) and above 0.3 (small correlation). Other correlations were categorized as follows: 0.3 < 0.5 = small correlation; 0.5 < 0.7 = moderate correlation; 0.7–0.9 = large correlation.

#### Criterion validity

Linear regression analyses were conducted to determine whether the consumption of healthy foods (fruit, vegetable and fish) and unhealthy foods (SSB, snacks) was linked to food literacy scores (range 1–5). From a conceptual point of view, average food literacy would be the logical independent variable and (un)healthy food intake the dependent variable in the analyses. However we chose the opposite as this allowed us to conduct a linear regression analyses (with food literacy scores as a continuous dependent variable), of which the coefficients (betas) were easy to interpret. First a crude model was run, followed by adjustments for expected confounders age, sex, educational level (low, medium, high), BMI (model 1), and additional adjustments for impulsiveness and self-control (model 2). Betas and 95% confidence intervals (CI) were obtained.

#### Capacity to distinguish

Linear regression analyses were conducted to determine the scales’ capacity to distinguish populations assumed to be high and lower in food literacy (registered dieticians vs. the general population). First a crude model was run in which ‘population’ (registered dieticians vs. general population) was the independent variable and the mean food literacy score (range 1–5) the dependent variable, followed by adjustments for age, sex, educational level (low, medium, high), BMI (model 1), and additional adjustments for impulsiveness and self-control (model 2). Betas and 95% confidence intervals (CI) were obtained.

## Results

### Participants

#### General adult population

The participants that completed the questionnaire (*n* = 755) were on average 44.8 (SD: 16.1) years old and the vast majority were women (90.7%) who had a healthy weight (61.4% (BMI 20–25 kg/m^2^)) and were highly educated (59.1%, Table 1).

**Table 1:** Participant characteristics of the sample of adults (*n* = 755) and registered dieticians (*n* = 207)

	Adult sample	Registered dieticians
Sex		
Women n (%)	685 (90.7%)	204 (98.5%)
Age		
± (SD)	44.8 (16.1)	43.4 (12.9)
Private n (%)	15 (2.0%)	4 (1.9%)
BMI^a^		
± (SD)	24.3 (4.4)	23.1 (3.31)
Private n (%)	94 (12.5%)	15 (7.2%)
BMI-category^a^ n (%)		
Obesity	61 (6.7%)	12 (5.8%)
Overweight	169 (18.6%)	26 (12.6%)
Healthy weight	406 (61.4%)	145 (70.0%)
Underweight	25 (2.7%)	6 (2.9%)
Private	94 (12.5%)	18 (8.7%)
Educational level n (%)		
Low	76 (10.2%)	–
Middle	228 (30.7%)	–
High	440 (59.1%)	207 (100%)
Private	11 (1.6%)	–
Ethnicity n (%)		
Dutch	735 (97.4%)	198 (95.6%)

^a^Based on self-reported body height and weight (kg/m^2^). Cut-off values BMI categories: underweight (< 18.5); healthy weight (18.5 < 25); overweight (25 < 30); obesity (> 30)

#### Registered dieticians

A total of 219 dieticians participated, of which 207 (94.5%) dieticians completed the questionnaire and were included in the analyses. The majority were women (93.2%) and were on average 43 years old (SD:12.9), with a total of 76.7% having a healthy weight. All the dieticians were highly educated (Table 1).

### Principal component analyses

The KMO measure of sampling adequacy was 0.88 and Bartlett’s Test was statistically significant (*p* < .000), indicating data adequacy for PCA. Moreover, none of the items showed a correlation above 0.90. PCA resulted in a total of 12 components with an eigenvalue above 1.0. The percentage of variance explained by these 12 components was 57%. However, the eigenvalue (1.30) of the eight components still exceeded the eigenvalue generated by means of the parallel analyses (1.27). Also the scree plot indicated eight rather than 12 components to be sufficient. In the next PCA which predefined the eight components the explained variance of the eight items dropped to 48%. Nevertheless seven items had commodities below 0.3 and were therefore removed. After rerunning the PCA the explained variance of the eight components increased again to 53%. However, six items did not show component-loadings or loaded on multiple components. After removing these items from a subsequent PCA, the explained variance increased to 56%. In the final PCA there were eight items which had a low loading on one of the eight components (< 0.5) and which were additionally removed. The final set consisted of 29 interrelated items divided over eight components and explaining 62% of the variance. Inspecting and interpreting the items belonging to each of the eight components, the following subthemes for each component were identified: 1) Food preparation skills; 2) Resilience and resistance; 3) Healthy snack styles; 4) Social and conscious eating; 5) Examining Food Labels; 6) Daily food planning; 7) Healthy budgeting and 8) Healthy food stockpiling; (Table 1). The scree plot, the pattern matrix and the correlation structure matrix of the final PCA are presented in Additional files 1, 2 and 3 (Table 2).

**Table 2:** List of the domains, items and Cronbach’s Alphas of the Self-Perceived Food Literacy Scale (29 items, α: 0.83)

ᅟI. Food preparation skills (6 items, α: 0.78)	
1. Are you able to prepare fresh vegetables in different ways? *For example cooking, steaming or stir frying, or in different dishes?*	
2. Do you find it difficult to prepare a meal with more than five fresh ingredients?	
3. Are you able to alter a recipe yourself? *For example if you are missing one of the ingredients?*	
4. Are you able to prepare fresh fish in different ways? *For example grilling, pan frying or stewing, or in different dishes?*	
5. Are you able to prepare a meal using fresh ingredients? *So without pre-packed and processed foods?*	
6. Are you able to see, smell or feel the quality of fresh foods? *For example of meat, fish or fruit?*	
ᅟII. Resilience and resistance (6 items, α = 0.80)	
7. Are you able to say ‘no’ to tasty snacks if you want to? *For example birthday treats or finger foods?*	
8. Imagine that you are at a place where you see and smell tasty foods. Are you able to resist the temptation of buying them? *For example at the train station, the petrol station, or at the bakery?*	
9. Are you able to eat healthily when you feel stressed?	
10. Do you choose foods that are in line with your mood? *For example if you are sad or annoyed?*	
11. Are you able to eat healthily if the situation deviates from a regular situation? *For example when you have unexpected guests or experience time pressure?*	
12. Do you eat the total contents of a bag or container of crisps, candies or cookies in one go?	
ᅟIII. Healthy snack styles (4 items, α = 0.58)	
13. Do you take along healthy snacks for yourself when you are on the go? *For example fruit, cherry-tomatoes, nuts?*	
14. Do you eat vegetables as snacks?	
15. Do you eat fruit as a snack?	
16. Do you have healthy snacks for yourself in stock? *For example nuts, carrots, cherry tomatoes, or mini cucumbers?*	
ᅟIV. Social and conscious eating (3 items, α = 0.69)	
17. Do you find it important to eat at the dinner table if you are eating with others?	
18. Do you find it important to eat dinner at the same time if you are with others?	
19. Do you engage in any other activities while eating? *For example reading, working, or watching television?*	
ᅟV. Examining food labels (2 items, α = 0.90)	
20. Do you compare the calories, fat, sugar or salt content of different products?	
21. Do you check the nutritional labels of products for calories, fat, sugar or salt content?	
ᅟVI. Daily food planning (2 items, α = 0.72)	
22. If you have something to eat, do you take account of what you will eat later that day?	
23. If you have something to eat, do you reflect on what you have eaten earlier that day?	
ᅟVII. Healthy budgeting (2 items, α = 0.85)	
24. Do you purchase healthy foods, even if they are a bit more expensive? *For example vegetables, fruit, or whole grain products?*	
25. Do you purchase healthy food, even if you have limited money? *For example vegetables, fruit, or whole grain products?*	
ᅟVIII. Healthy food stockpiling (4 items, α = 0.81)	
26. Do you have 4 or more packages of crisps, pretzels or savoury snacks in stock?	
27. Do you have 4 or more packages of candy, cookies or chocolate in stock?	
28. Do you have 4 or more bottles of sugar sweetened beverages or lemonade with sugar in stock?	
29. Do you have 4 or more cartons of fruit juice in stock?	

### Internal consistency

The internal consistency of the overall scale was considered good (Cronbach’s α = 0.83). Four components showed a good reliability with Cronbach’s α varying from 0.80–0.90 and three components showed adequate reliability with Cronbach’s α varying from 0.68–0.78, Table 1. Only one subscale (‘healthy snack styles’) had a lower than sufficient (0.7) Cronbach’s alpha of 0.58, indicating that this subscale in isolation showed inadequate reliability.

### Convergent and divergent validity

Bivariate correlational analyses revealed a consistent pattern of correlations between the SPFL and self-control and impulsiveness. Self-control and food literacy were positively correlated to a moderate magnitude (*r* = 0.51, *p* = <.001) where impulsiveness and food literacy were negatively correlated to a small extent (*r* = − 0.31, *p* = <.01). These findings confirm convergent and divergent validity with respect to psychosocial individual traits associated with (un)healthy food choices.

### Criterion validity

As theoretically predicted, higher food literacy was associated with higher frequency of fruit (≥5 times/week), vegetables (≥5 times/week) and fish (≥1 times/week) consumption and those who reported consuming larger portions of fruit (≥ 2 pieces/day) and vegetables (≥ 200 g/day) a day in comparison with participants who consumed lower amounts (Table 3). For example participants who consumed fruit every day were more food literate than those who consumed fruit fewer than four times a week (B = 0.28, SE = 0.04, *t* = 7.71, 95%-CI = 0.21 to 0.35). Participants who consumed at least the recommended 250 g of vegetables per day were also more food literate than participants who consumed 150 g or less vegetables per day (B = 0.28, SE = 0.03, *t* = 9.83, 95%-CI = 0.22 to 0.34).

**Table 3:** Results of the linear regression analyses for the associations between healthy food consumption (categorical variables) and the self-perceived food literacy scale (continuous variable) of Dutch adults (*n* = 755)

	Crude model^1^					Model 1^1^					Model 2^1^				
	*B* ^*2*^	*SE*	*T*	*95%CI*		*B* ^*2*^	*SE*	*t*	*95%CI*		*B* ^*2*^	*SE*	*t*	*95%CI*	
Vegetables (# days per week)															
2–4 days*	(ref)					(ref)					(ref)				
5–6 days	0.19	0.06	3.42	0.08	0.31	0.22	0.06	3.39	0.09	0.34	0.22	0.05	3.81	0.11	0.34
Everyday	0.50	0.05	9.35	0.40	0.61	0.49	0.06	8.10	0.37	0.61	0.45	0.05	8.23	0.34	0.56
Vegetables (amount per day)															
50–150 g (1–3 serv. spoons)	(ref)					(ref)					(ref)				
200 g (4 serv. spoons)	0.16	0.03	4.87	0.09	0.22	0.16	0.04	4.39	0.09	0.22	0.14	0.03	4.42	0.08	0.20
≥ 250 g (≥5 serv. spoons)	0.36	0.03	11.97	0.30	0.42	0.35	0.03	9.38	0.29	0.41	0.28	0.03	9.83	0.22	0.34
Fruit (# days per week)															
0–4 days	(ref)					(ref)					(ref)				
5–6 days	0.19	0.02	4.36	0.10	0.27	0.17	0.05	3.61	0.08	0.26	0.16	0.04	3.84	0.08	0.24
Everyday	0.38	0.04	10.31	0.30	0.45	0.34	0.04	8.38	0.26	0.41	0.28	0.04	7.71	0.21	0.35
Fruit (amount per day)															
≤ 1 piece	(ref)					(ref)					(ref)				
2 pieces	0.20	0.03	6.08	0.14	0.27	0.20	0.04	5.61	0.13	0.27	0.20	0.03	6.22	0.14	0.26
≥ 3 pieces	0.33	0.04	8.15	0.25	0.41	0.31	0.04	7.06	0.23	0.40	0.25	0.04	6.21	0.17	0.33
Fish (# days per week)															
< 1 day or never	(ref)					(ref)					(ref)				
1 day	0.14	0.03	4.47	0.08	0.21	0.14	0.04	3.93	0.07	0.20	0.12	0.03	3.95	0.06	0.18
≥ 2 days	0.25	0.03	7.55	0.19	0.32	0.25	0.04	6.72	0.18	0.32	0.20	0.03	5.90	0.13	0.27

^1^Model 1: crude model, model 2: adjusted for age, sex, educational level, BMI, model 3: adjusted for age, sex, educational level, BMI, impulsiveness and self-control

^2^Five-point Likert scale

*None of the participants consumed vegetables on fewer than 2 days a week . ^~^ non-significant

In addition, participants who never/rarely consumed SSB, large snacks or small snacks also possessed a higher food literacy in comparison with participants who consume larger quantities of these unhealthy products (Table 4). For example, participants who consumed large snacks at least four times a week had lower food literacy compared to participants who rarely/never consumed large snacks (B = − 0.47, SE = 0.04, *t* = − 10.79 95%-CI = − 0.56 to − 0.39). Participants who drank 2 or more glasses of SSB per day also had a lower food literacy compared to individuals who never/rarely drank SSB (B = − 0.23, SE = 0.04, *t* = − 5.60, 95%-CI = − 0.31 to − 0.15).

**Table 4:** Results of the linear regression analyses for the associations between unhealthy food consumption (categorical variables) and self-perceived food literacy (continuous variable) of Dutch adults (*n* = 755)

	Model 1^1^					Model 2^1^					Model 3^1^				
	*B* ^2^	*SE*	*T*	*95%CI*		*B* ^2^	*SE*	*t*	*95%CI*		*B* ^2^	*SE*	*t*	*95%CI*	
Sugar sweetened beverages (# days per week)															
Never/rarely (=1-3d/month)	(ref)					(ref)					(ref)				
1–3 days	−0.18	0.04	−4.74	−0.25	− 0.10	− 0.12	0.04	−3.0	− 0.20	− 0.04	− 0.10	0.04	−2.72	− 0.17	−0.03
4–7 Days	− 0.26	0.05	− 5.6	− 0.35	−0.17	− 0.19	0.05	−3.5	− 0.29	− 0.08	− 0.18	0.05	−3.74	− 0.27	− 0.08
Sugar sweetened beverages (amount per day)															
None-less than 1 glass	(ref)					(ref)					(ref)				
1 glass	−0.15	0.03	−5.04	− 0.20	− 0.09	− 0.13	0.03	−3.90	− 0.19	−0.06	− 0.08	0.03	−2.81	− 0.14	−0.03
2 or more glasses	−0.36	0.04	−9.03	−0.43	−0.28	− 0.29	0.05	−6.57	− 0.38	−0.21	− 0.23	0.04	−5.60	− 0.31	−0.15
Small snacks (# days per week)															
Never/rarely (=1-3d/month)	(ref)					(ref)					(ref)				
1 portion	−0.15	0.04	−3.80	− 0.23	−0.07	− 0.11	0.05	−2.47	− 0.20	−0.02	− 0.08	0.04	−2.03	− 0.16	−0.003
2 or more portions	−0.15	0.04	−4.09	−0.22	−0.08	− 0.15	0.04	−3.75	− 0.23	−0.07	− 0.11	0.04	− 3.07	− 0.18	−0.04
Small snacks (amount per day)															
None-less than 1 portion	(ref)					(ref)					(ref)				
1 portion	−0.08	0.05	−1.76	−0.17	0.009^~^	−0.10	0.05	−1.81	−0.20	0.008^~^	−0.10	0.05	−2.05	−0.20	−.004
2 or more portions	−0.30	0.04	−6.97	−0.39	−0.22	− 0.28	0.05	−5.40	− 0.38	−0.18	− 0.20	0.05	−4.27	− 0.30	−0.11
Large snacks (# days per week)															
Never/rarely (=1-3d/month)	(ref)					(ref)					(ref)				
1–3 days	−0.23	0.03	−8.41	− 0.28	−0.18	− 0.19	0.03	−6.10	− 0.25	−0.13	− 0.19	0.03	−6.10	− 0.25	−0.13
4 or more days	−0.50	0.04	−12.79	−0.58	−0.43	− 0.47	0.04	−10.79	− 0.56	−0.39	− 0.47	0.04	−10.79	− 0.56	−0.39
Large snacks (amount per day)															
None-less than 1 portion	(ref)					(ref)					(ref)				
1 portion	−0.24	0.03	−7.91	−0.30	−0.18	− 0.20	0.04	−5.69	− 0.27	−0.13	− 0.15	0.03	−4.71	− 0.22	−0.09
2 or more portions	−0.52	0.04	−12.79	−0.60	−0.44	− 0.42	0.05	−8.82	− 0.51	−0.33	− 0.31	0.04	−6.98	− 0.40	−0.22

^1^Model 1: crude model, model 2: adjusted for age, sex, educational level, BMI, model 3: adjusted for age, sex, educational level, BMI, impulsiveness and self-control

^2^ Five-point Likert scale. ^~^ non-significant

Moreover, the figures for both the association between a higher food literacy and healthier food consumption indicated a dose-response association. For example, compared to participants who consumed vegetables < 4 days a week, participants who ate vegetables 5–6 times a week had higher food literacy scores (B = 0.22, SE = 0.05, *t* = 3.81, 95%-CI = 0.11 to 0.34) whereas this was even higher among participants who consumed vegetables every day (B = 0.45, SE = 0.05, *t* = 8.23, 95%-CI = 0.34 to 0.56, Table 3). With the exception of small snacks, a dose-response association was also noticeable for lower food literacy levels and unhealthy food consumption.

### Capacity to distinguish

On average the adult population recruited in this study scored 3.83 (SD = 0.41) on the SPFL scale whereas the mean score of dieticians was higher 3.99 (SD = 0.30) on a scale of one to five. Linear regression analyses showed small, but statistically significant, differences between both groups (B = 0.16, SE = 0.03, *t* = 5.60, 95%-CI = 0.11 to 0.22) that remained statistically significant after adjustments for age, sex, educational level and BMI (B = 0.10, SE = 0.03, *t* = 3.12, 95%-CI = 0.04 to 0.17) and additionally when impulsiveness and self-control were added to the model (B = 0.08, SE = 0.03, *t* = 2.83, 95%-CI = 0.03 to 0.14).

## Discussion

A 29 item scale was developed to measure self-perceived food literacy with respect to healthy eating among an adult population. The SPFL scale covered eight domains of food literacy, namely: Food preparation skills, Resilience and resistance, Healthy snack styles, Social and conscious eating, Examining Food Labels, Daily food planning, Healthy budgeting and Healthy food stockpiling. The overall scale and seven out of eight subscales showed good reliability. However, one subscale (Healthy snack manners) showed inadequate reliability and is therefore not acceptable for use in isolation. The overall SPFL scale was positively correlated with self-control and negatively correlated with impulsivity, indicating its convergent and divergent validity. Moreover, criterion validity was revealed, indicating that self-perceived food literacy was positively associated with healthy food consumption and negatively with unhealthy food consumption. In conclusion, the scale was able to distinguish an assumable higher food literate population, in other words dieticians, from the general adult population, although the distinctions were rather small and we should question the clinical relevance.

Our study is one of the first to develop and evaluate a comprehensive measure to assess individuals’ self-perceived food literacy with respect to healthy eating. Recently a 12 item short food literacy questionnaire (SFLQ) was developed based on a Swiss adult population with the aim being to determine individual skills and abilities needed for healthy food choices [26]. Moreover, a study in Taiwan constructed nutrition literacy indicators based on a Delphi consensus study [27]. Although the aims of our studies were identical, the items surprisingly differed from our SPFL scale. The SFLQ concerns peoples’ understanding and search capacities of information about a healthy diet and their ability to judge and use this information. The SFLQ closely aligns with the concepts of functional, interactive and critical health literacy [29, 55] whereas our SPFL scale includes a wider range of food literacy practicalities, oriented towards the food literacy concept definition of Vidgen et al. [14]. The scales may be used synergistically in future research to get a better understanding of the similarities or discrepancies of both scales in behavioral nutrition research. A scoping review on food literacy including 19 papers was also published recently [11]. This review revealed that individual skills related to food selection, preparation, handling and storage were fundamental for the concept of food literacy. Efficacy, confidence and capability related to food, nutrition and preparations in difficult situations were also emphasized. Although the review was published after the development of our scale, it is reassuring to know that the SPFL scale does cover most essential attributes of individual food literacy that were emphasized by the review.

In this study we only determined the associations between overall self-perceived food literacy and (un)healthy food consumption. As indicated the SPFL scale covers eight domains and a deeper understanding of the interplay between the self-perceived food literacy domains and its association with (un)healthy eating and differences between subgroups (e.g. age, sex) would be a sensible next step. It would also be interesting to ascertain which other individual, social or contextual factors may enable (e.g. spare time) or inhibit (e.g. financial stress) food literacy in order to get a better understanding of external circumstances that interfere with the potential of being food literate. Moreover, future research may expend the health focus of self-perceived food literacy measurements as other food literacy related concepts like sustainability, trade and economics are important but not stressed by the SPFL scale. Finally it is important to understand to what extent food literacy contribute to people’s health.

Although we developed a comprehensible valid scale driven by theory [28, 29] and expert views, and without undermining the importance of the present work, we acknowledge that a quantitative questionnaire is a rather limited way of assessing the complex concept of food literacy. Food literacy consists of a wide variety of subtle, dynamic features that differ in situations and over a person’s life [9, 12]. Its comprehensibility and context dependence makes quantitative measurement of food literacy challenging [56]. Besides that food literacy includes additional themes beyond individual behavioral nutrition and healthy food choices, outlined in a recently published scoring review which analysed a large number of studies into food literacy (*n* = 67). Themes included skills/behavior, food/health choices, culture, knowledge, emotions and food systems. Moreover, the capability of households, communities or nations to protect diet quality through dietary resilience beyond the individual food literacy domains (e.g. infrastructural or cultural aspects) is likely to play a role in food literacy, but is difficult to capture by means of a standardized questionnaire, making it difficult to achieve a unifying understanding of people’s food literacy [11]. During our expert meeting and in prior research [14, 26] similar challenges were indeed emphasized. Future research should investigate measurements beyond the individual scope, including the capability of households, communities or nations to protect diet quality through dietary resilience. Although quantitative measures are required and important, we may also broaden our view and learn from other scientific fields like anthropology and sociology in order to gain a better understanding of food literacy among individuals using different measurement or monitoring techniques.

The strengths of this study include the large study sample, the mixed-method approach, comprising an expert and theory-driven scale development and the determination of convergent, divergent and criterion validation, as well as the capacity to distinguish by using quantitative analyses. The limitations of the study include its cross-sectional character and the lack of representativeness of the Dutch adult population included in the study. The sample had a higher education on average and comprised more women than the general Dutch population. Besides that the participants in the adult population were recruited via the Netherlands Nutrition Centre. Potentially this may account for an underestimation of the scale’s capacity to distinguish between the dieticians and the ‘general’ adult population, as this population is likely to have a higher interest in nutrition than the general Dutch population who do not follow this Centre on social media. The final limitations of the study were that all outcome measures were self-reported and both food literacy and food intake were measured successively in the same questionnaire. This may have caused bias by expressing participants’ ideas rather than their actual behavior and because responses to the food intake questions were primed by the food literacy items earlier in the questionnaire and were therefore more in accordance with their early answers [57]. However, these are common limitations in the field of behavioral nutrition research which uses surveys that measure behavioral determinants and dietary behavior.

The results of this study confirmed the criterion validity of the SPFL scale and showed that food literacy is positively associated with healthy food intake and negatively with unhealthy food intake. Besides using this scale in scientific research, the tool can also be used outside academia. Dieticians could potentially use the tool as a screening instrument to determine and monitor an individual’s food literacy over time. Moreover, this study showed the importance of the collection of interrelated knowledge, skills and behaviors required to plan, manage, select, prepare and eat food to meet the needs for a healthy food intake. National public health nutrition programmes should incorporate the broad range of practicalities that comprise food literacy and/or add this to an approach that focuses primarily on improving nutritional knowledge towards healthy eating. However, such programmes should always be accompanied by efforts to create healthier food environments that enable individuals to implement their food literacy.

## Conclusion

The 29 item SPFL scale is a validated, expert-based and theory-driven tool for measuring self-perceived food literacy with respect to healthy eating among adults. Higher levels of food literacy were associated with more self-control, less impulsiveness and healthier food consumption. The SPFL scale can be used in a range of studies, including in an evaluation of interventions aimed at improving individual food literacy. Additional research is needed to validate the SPFL scale in different populations (different age groups, socioeconomic groups, male population) and in different contexts and settings and future studies could emphasise food literacy measures beyond individual dietary behavior.

## Additional files

Additional file 1: Component Correlation Matrix. (DOCX 15 kb)

Additional file 2: Scree plot. (DOCX 27 kb)

Additional file 3: Pattern matrix. (DOCX 22 kb)
