# Supplementary material for: Towards the measurement of food literacy with respect to healthy eating: the development and validation of the self perceived food literacy scale among an adult sample in the Netherlands
Source: Int J Behav Nutr Phys Act. 2018 Jun 18;15:54. doi: 10.1186/s12966-018-0687-z (PMC6006995; doi:10.1186/s12966-018-0687-z)
Supplement: Supplementary file 1 — Component Correlation Matrix. (DOCX 15 kb) [file 12966_2018_687_MOESM1_ESM.docx]

**Additional file 1. Component Correlation Matrix**

| **Component Correlation Matrix** | | | | | | | | |
| --- | --- | --- | --- | --- | --- | --- | --- | --- |
| Component | 1 | 2 | 3 | 4 | 5 | 6 | 7 | 8 |
| 1 | 1,000 | -,196 | ,148 | -,096 | ,191 | ,197 | -,314 | -,312 |
| 2 | -,196 | 1,000 | -,033 | ,178 | -,156 | -,226 | ,207 | ,153 |
| 3 | ,148 | -,033 | 1,000 | ,027 | -,053 | ,154 | -,154 | -,067 |
| 4 | -,096 | ,178 | ,027 | 1,000 | -,032 | -,177 | ,112 | ,184 |
| 5 | ,191 | -,156 | -,053 | -,032 | 1,000 | ,015 | -,117 | -,070 |
| 6 | ,197 | -,226 | ,154 | -,177 | ,015 | 1,000 | -,164 | -,263 |
| 7 | -,314 | ,207 | -,154 | ,112 | -,117 | -,164 | 1,000 | ,164 |
| 8 | -,312 | ,153 | -,067 | ,184 | -,070 | -,263 | ,164 | 1,000 |
| Extraction Method: Principal Component Analysis.  Rotation Method: Oblimin with Kaiser Normalization. | | | | | | | | |
