# Supplementary figures and images for: Towards the measurement of food literacy with respect to healthy eating: the development and validation of the self perceived food literacy scale among an adult sample in the Netherlands
Source: Int J Behav Nutr Phys Act. 2018 Jun 18;15:54. doi: 10.1186/s12966-018-0687-z (PMC6006995; doi:10.1186/s12966-018-0687-z)

**Additional file 2. Scree plot**
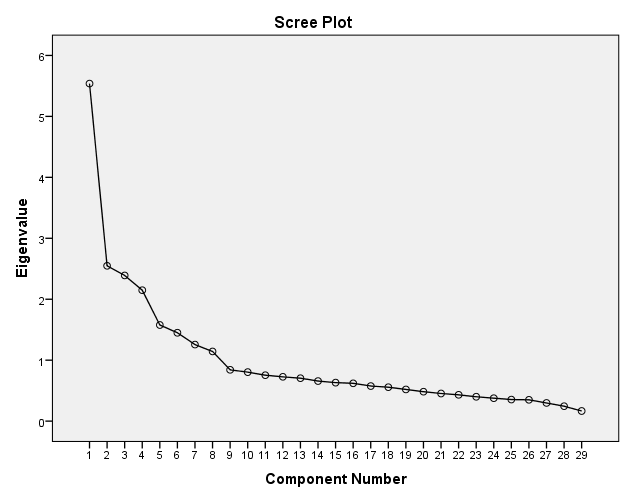

Supplement: Supplementary file 2 — Scree plot. (DOCX 27 kb) [file 12966_2018_687_MOESM2_ESM.docx]
