# Supplementary material for: Towards the measurement of food literacy with respect to healthy eating: the development and validation of the self perceived food literacy scale among an adult sample in the Netherlands
Source: Int J Behav Nutr Phys Act. 2018 Jun 18;15:54. doi: 10.1186/s12966-018-0687-z (PMC6006995; doi:10.1186/s12966-018-0687-z)
Supplement: Supplementary file 3 — Pattern matrix. (DOCX 22 kb) [file 12966_2018_687_MOESM3_ESM.docx]

**Additional file 3. Pattern matrix**

|  | **COMPONENT - LOADING** | | | | | | | |
| --- | --- | --- | --- | --- | --- | --- | --- | --- |
|  | 1 | 2 | 3 | 4 | 5 | 6 | 7 | 8 |
| Are you able to prepare fresh vegetables in different ways?  *For example, cooking, steaming or stir frying or in different dishes.* | ,790 |  |  |  |  |  |  |  |
| Do you find it difficult to prepare a meal with more than five fresh ingredients? | ,777 |  |  |  |  |  |  |  |
| Are you able to alter a recipe yourself?  *For example, if you are missing one of the ingredients*. | ,768 |  |  |  |  |  |  |  |
| Are you able to prepare fresh fish in different ways?  *For example, grilling, pan frying or stewing , or in different dishes* | ,667 |  |  |  |  |  |  |  |
| Are you able to prepare a meal using fresh ingredients?  *So without pre-packed and processed foods?* | ,621 |  |  |  |  |  |  |  |
| Are you able to see, smell or feel the quality of fresh foods?  *For example of meat, fish or fruit* | ,536 |  |  |  |  |  |  |  |
| Do you have 4 or more packages of crisps, pretzels or savory snacks in stock ? = |  | ,831 |  |  |  |  |  |  |
| Do you have 4 or more packages of candy, cookies or chocolate in stock? |  | ,799 |  |  |  |  |  |  |
| Do you have 4 or more bottles of sugar sweetened beverages or lemonade with sugar in stock? |  | ,773 |  |  |  |  |  |  |
| Do you have 4 or more cartons of fruit juice in stock? |  | ,721 |  |  |  |  |  |  |
| Are you able to say 'no' to tasty snacks if you want to?  *For example birthday treats or finger foods* |  |  | ,711 |  |  |  |  |  |
| Imagine that you are at a place where you see and smell tasty foods. Are you able to resist the temptation of buying this?  *For example at the train, the petrol station or at the bakery?* |  |  | ,687 |  |  |  |  |  |
| Are you able to eat healthy when you feel stressed? |  |  | ,675 |  |  |  |  |  |
| Do you choose foods that are in line with your mood?  *For example if you are sad or annoyed?* |  |  | ,647 |  |  |  |  |  |
| Are you able to eat healthy if the situation deviates from a regular situation?  *For example, when you have unexpected guests or experience time pressure* |  |  | ,620 |  |  |  |  |  |
| Do you eat the total amount of a package or container of crisps, candies or cookies at once? |  |  | ,617 |  |  |  |  |  |
| Do you bring healthy snacks for yourself when you are on the go?  *For example, fruit, cherry-tomatoes, nuts* |  |  |  | -,743 |  |  |  |  |
| Do you eat vegetable as snacks? |  |  |  | -,708 |  |  |  |  |
| Do you eat fruit as a snack? |  |  |  | -,679 |  |  |  |  |
| Do you have healthy snacks for yourself in stock?  *For example nuts, carrots, cherry-tomatoes, or mini-cucumbers* |  |  |  | -,581 |  |  |  |  |
| Do you find it important to eat at the dinner table if you are eating with others? |  |  |  |  | ,885 |  |  |  |
| Do you find it important to eat dinner at the same time if you are with others? |  |  |  |  | ,812 |  |  |  |
| Are you involved in other activities while eating?  *For example reading, working or watching television?* |  |  |  |  | ,662 |  |  |  |
| Do you compare the calories, fat, sugar or salt content of different products? |  |  |  |  |  | ,962 |  |  |
| Do you check nutrition fact labels of products for calories, fat, sugar or salt content? |  |  |  |  |  | ,951 |  |  |
| If you have something to eat, do you consider what you will eat later that day? |  |  |  |  |  |  | ,922 |  |
| If you have something to eat, do you reflect on what you have eaten before on that day? |  |  |  |  |  |  | ,909 |  |
| Do you purchase healthy foods, even if it is a bit more expensive?  For example, vegetables, fruit or whole grain products |  |  |  |  |  |  |  | -,784 |
| Do you purchase healthy food, even if you have limited money?  For example, vegetables, fruit or whole grain products |  |  |  |  |  |  |  | -,755 |
| Extraction Method: Principal Component Analysis.  Rotation Method: Oblimin with Kaiser Normalization. | | | | | | | | |
| a. Rotation converged in 9 iterations. | | | | | | | | |
